# Increased emergency room visits without corresponding rehospitalizations in cannabis users with psychosis

**DOI:** 10.1192/j.eurpsy.2024.799

**Published:** 2024-08-27

**Authors:** O. Martin-Santiago, M. Calvo-Valcarcel, P. Martinez.Gimeno, C. Alario-Ruiz, B. Arribas-Simon

**Affiliations:** ^1^Hospital Clinico Universitario, Valladolid, Spain

## Abstract

**Introduction:**

Epidemiological studies have established a complex relationship between cannabis consumption and a heightened risk of psychotic disorders, including schizophrenia. However, this connection is multifaceted, influenced by genetics, environment, and individual psychology. Surprisingly, despite a surge in emergency room (ER) visits associated with cannabis consumption and psychosis, there haven’t been significant increases in hospital readmissions. This rise in ER visits can be attributed to the increasing social acceptance of cannabis and its legalization in some regions, increasing the likelihood of adverse effects. Furthermore, the higher potency of contemporary cannabis can trigger psychotic reactions, particularly in those consuming elevated levels of THC, its primary psychoactive component.

**Objectives:**

This study aimed to compare the rates of readmissions and ER visits one year after hospital discharge among patients diagnosed with schizophrenia and other psychotic disorders, stratified by cannabis consumption.

**Methods:**

We collected sociodemographic and clinical data from 109 patients after discharge from a psychiatric hospitalization unit.

**Results:**

Patients who consumed cannabis (N=35) were younger than non-consumers (M=31.4; SD=10.0 vs M=44.3; SD=11.4; t(107)=5.71; p<.01), with no significant gender differences, hospital stay durations, or proportions of schizophrenia diagnoses (33.3%). The readmission rates and time to readmission were similar between both groups. Interestingly, 54.2% of cannabis consumers required emergency care (*X^2^*_(1, N = 109)_= 4.1, p = .04), with 73.6% not needing admission (*X^2^*_(1, N = 109)_= 5.5, p = .01), in contrast to 33.7% and 56% in the non-consumer group. The time to the first urgent care visit was shorter in the consumer group (M=59.5; SD=56.3) compared to the non-consumer group (M=105.8; SD=93.1; t_(107)_=1.92; p=.03).

**Conclusions:**

This study reveals that patients with psychosis and cannabis consumption tend to visit ER services more frequently despite utilizing fewer hospital resources like hospitalizations. Notably, despite the increased ER visits, there hasn’t been a corresponding rise in hospital readmissions. These would be due to individuals experiencing cannabis-related psychotic episodes receiving suitable assessment and treatment in the ER, obviating the need for prolonged hospitalization. Furthermore, some psychotic episodes may naturally resolve over time, particularly with reduced or discontinued cannabis consumption. Our result highlights the need for personalized care approaches targeting this group, effectively addressing acute episodes related to cannabis use and psychosis. Addressing this trend requires a multidisciplinary approach involving mental health professionals, addiction specialists, and emergency response teams.

**Disclosure of Interest:**

None Declared

